# Green synthesis revolution: harnessing biological factories for sustainable insect pheromone production in agriculture

**DOI:** 10.3389/fpls.2026.1811832

**Published:** 2026-07-07

**Authors:** Ke-Xin Wang, Yi-Han Xia

**Affiliations:** School of Agriculture and Biotechnology, Sun Yat-sen University, Shenzhen, China

**Keywords:** insect pheromone, integrated pest management, lipid metabolic engineering, plant factory, yeast cell factory

## Abstract

Insect sex pheromones enable species-specific monitoring and mating disruption in integrated pest management, but wider adoption is limited by manufacturing complexity, particularly for molecules requiring stringent control of chain length, double-bond position/geometry, and terminal functional groups. Synthetic biology and lipid metabolic engineering have enabled microbial and plant “biological factories” to produce fatty-acid-derived moth pheromone components from renewable carbon. This review organizes pheromone biomanufacturing around structure-defining biochemical decision points and the corresponding enzyme modules, summarizes the current gene pool (desaturases, reductases, β-oxidation–linked chain shortening, and terminal conversions), and discusses key host constraints that govern product titer, product spectrum, and host fitness, including precursor supply, competing sinks, redox balance, compartmentalization, and product toxicity. We compare yeast and plant platforms and synthesize lessons from representative case studies, highlighting recurring engineering strategies and current limitations. Finally, we outline priorities for improving predictability and scalability, including expanding enzyme selectivity/coverage and implementing standardized benchmarking supported by modern genome engineering and data-driven design.

## 1 Introduction

Insects use semiochemicals to coordinate a wide range of behaviors within and between species. Pheromones are a subset of these signals: compounds released by an individual and detected by conspecifics, triggering stereotyped behavioral and/or physiological responses. Depending on context, pheromones are commonly classified as sex, alarm, aggregation (and anti-aggregation), oviposition-deterring/epideictic, and trail pheromones (Tewari et al., 2014).

Owing to their high species specificity and activity at very low doses, pheromones have become important tools in integrated pest management (IPM) (Witzgall et al., 2010; Tewari et al., 2014). They are widely used for surveillance (monitoring and decision support) and, for selected targets, for population suppression through mating disruption, mass trapping, and attract-and-kill approaches (Tewari et al., 2014; Anugwom and Audi, 2026). In many cases, field performance is determined as much by product specifications—blend composition, stereochemical purity, release kinetics, and dispenser or formulation design—as by the identity of the active ingredient(s) (Sisay et al., 2025). These requirements link pheromone biology directly to manufacturing and downstream product development.

At the same time, well-recognized limitations of insecticide-centered control, including resistance evolution, pest resurgence, and residue concerns, have increased interest in behavior-based alternatives and complements within IPM. Among semiochemicals-based tactics, sex pheromone-mediated mating disruption is one of the most widely implemented in practice (Thiery et al., 2023; Anukiruthika and Jayas, 2025). Pheromone-based approaches can reduce selection pressure on insecticide modes of action and limit off-target effects, but large-scale adoption still depends on cost, deployment logistics, and reliable performance under field conditions. These constraints motivate continued innovation not only in how pheromones are deployed, but also in how they are produced.

Natural pheromone titers in insects are extremely low, so chemical synthesis remains the dominant route for supplying material for research and field use. However, routes developed at laboratory scale do not always translate into cost-efficient, large-volume manufacture, particularly for blends that require stringent control of double-bond position/geometry and stereochemical purity (Turczel et al., 2018). This has driven the development of biotechnology-enabled production platforms (“cell and plant factories”), in which pheromone components are generated from renewable carbon through engineered lipid metabolism and tailored enzyme modules. Recent advances in yeast and plant engineering provide a growing toolbox for producing fatty-acid-derived moth pheromones, while also raising practical questions about purification, standardization, formulation, and controlled release.

Relevant literature was identified through searches in Web of Science, PubMed, and Google Scholar using keywords related to insect pheromone biosynthesis and metabolic engineering, limited to studies published up to 2025, and supplemented by citation tracking. Studies were included if they reported experimental work on pheromone biosynthesis, enzyme characterization, or metabolic engineering in microbial or plant systems. In this review, we summaries progress in the biotechnological production of fatty-acid-derived insect sex pheromones from a lipid biosynthesis and synthetic biology perspective. We first describe the key biochemical decision points that define pheromone structures—chain length, double-bond position and geometry, and terminal functional groups—and use these to organize the available enzyme toolkits. We then discuss how these modules have been assembled and optimized in representative hosts, with emphasis on precursor supply, competing pathways, compartmentalization, and product toxicity as recurring engineering constraints. Finally, we identify limitations in the current toolkit—such as restricted regio-/stereoselectivity, incomplete coverage of functional-group conversions, and context-dependence across hosts—and outline practical directions for improvement, including enzyme discovery and engineering, pathway balancing and dynamic control, and standardized characterization that enables comparison across platforms.

## 2 Biological factories for pheromone production

### 2.1 Pheromone biosynthesis in insect

Most commercially used lepidopteran sex pheromones are derivatives of C10–C18 fatty acids. In moth pheromone glands, palmitic (16:0) or stearic acid (18:0) is desaturated by lineage−specific fatty−acyl desaturases (FADs) (often Δ11, Δ9, Δ10, Δ14, or multifunctional desaturases) to establish double−bond position and geometry. Chain length is then adjusted by β−oxidation or, less frequently, elongation. The resulting acyl−CoAs are converted to alcohols by fatty−acyl reductases (FARs), to aldehydes via alcohol oxidation or direct routes, and to acetates by acetyltransferases.

Beyond Lepidoptera, other insect groups employ chemically distinct pheromone classes. In several Diptera, long-chain hydrocarbons derived from fatty-acid metabolism contribute to pheromonal communication, typically via chain elongation followed by reduction and decarboxylation/decarboxylation steps that yield unsaturated hydrocarbons (Jurenka, 2004). In addition, many aphids use isoprenoid alarm pheromones such as (E)-β-farnesene; biosynthesis proceeds via IPP/DMAPP generation (mevalonate pathway in many insects), formation of farnesyl diphosphate by farnesyl diphosphate synthase, and conversion to the volatile product by terpene synthases (Verheggen et al., 2010). Notably, for some aphid species, the identity of the enzyme(s) catalyzing the final terpene-forming step remains unresolved (Witzgall et al., 2010; Vandermoten et al., 2012).

### 2.2 The gene pool available for pheromone biosynthesis

With these decision points in mind, we next consider the enzyme repertoire that has been validated to implement them in heterologous systems. The high cost of synthetic pheromones makes their widespread application in low-value crops difficult. Then, biological factory built based on biotechnology have inspired the development. In the factory, target compounds can be efficiently produced from cheap renewable materials through metabolic engineering and biotransformation techniques.

Functionally validated FADs and FARs have been isolated from diverse moth taxa, but their practical utility is often constrained by selectivity and strong context dependence in heterologous systems. For desaturases, only a subset provides the regio- and stereoselectivity required to install the correct double-bond position and geometry without generating undesired isomers, and many enzymes are partially promiscuous with respect to chain length or substrate saturation state. FARs show similar limitations: some are highly selective, whereas others reduce a broader range of acyl-CoA substrates, leading to mixed alcohol products or reduced flux toward the desired pheromone intermediate. In addition, Chain-shortening strategies based on β-oxidation can also be effective, but their outputs are frequently host- and pathway-dependent because β-oxidation is tightly integrated with native lipid homeostasis.

A major reason for this variability is that these enzymes evolved in the biochemical context of insect pheromone glands and often perform differently when transferred into yeast or plant hosts. For membrane-bound desaturases, activity can be altered by differences in endoplasmic reticulum membrane composition (Ding et al., 2014; Xia et al., 2022), protein folding and insertion efficiency, cytochrome b5-dependent electron transfer (Forman et al., 2024), and the availability and composition of host acyl-CoA pools (Zhou et al., 2016). In addition, heterologous hosts differ in redox balance, compartmentation, and endogenous lipid flux, all of which can shift substrate competition, cofactor availability, and host background metabolism, so the same enzyme may generate different product spectra or efficiencies in different chassis. As a result, the current “gene pool” is best viewed not as a simple parts list, but as a set of enzyme variants whose usefulness depends on the target pheromone structure and the biochemical context of the chosen host.

**Table 1**, **2** therefore summarize the currently functionally characterized enzymes as functional resource maps, highlighting where coverage is relatively strong, particularly for desaturases and reductases associated with common Type I moth pheromone scaffolds, and where important gaps remain, including unusual chain lengths, rare regio-/stereochemical outcomes, and certain terminal conversion steps. Addressing these bottlenecks increasingly relies on combining enzyme discovery and comparative characterization with protein engineering strategies, including active-site mutagenesis, domain-level optimization, and subcellular retargeting. More recently, structure-guided approaches and AI-assisted prediction have begun to emerge as promising tools for prioritizing candidate enzymes, identifying residues linked to regio-/stereoselectivity, and accelerating the improvement of catalytic performance in heterologous systems (Lu et al., 2025). Collectively, these efforts are helping shift the field from descriptive enzyme cataloging toward a more predictive and design-oriented toolkit for pheromone biomanufacturing.

**Table 1:** Functionally characterized fatty acyl desaturases and acyl-CoA oxidases involved in insect sex pheromone biosynthesis.

Gene ID/ protein accession	Gene name	*Species*	Functional classification	Product	Reference
13430288	Cricdes3	*Acheta domesticus*	Δ9	Z9-16:acid, Z9-18:acid	(Hu et al., 2020)
163311887	AdD12DES	*A. domesticus*	Δ12	(Z,Z)-9,12-16:acid, (Z,Z)-9,12-18:acid	(Petkevicius et al., 2022)
309753140	Ape-PGΔ11	*Antheraea pernyi*	Δ11	Z11-16:acid	(Wu et al., 2014)
309753142	Ape-PGΔ6	*A. pernyi*	Δ6	E6-16:acid	(Wu et al., 2014)
16265793	RBLRG-Z/E 11	*Argyrotaenia velutinana*	Δ11	Z11-14:acid, E11-14:acid	(Li et al., 2023)
7271958	RBLRFB-Z9	*A. velutinana*	Δ9	Z9-16:acid, Z9-18:acid	(Li et al., 2023)
112050801	Ban-Δ11	*Bicyclus anynana*	Δ11	Z11-16:acid	
/	Δ9-Bter, Δ9-Bluc, Δ9-Blap	*Bombus terrestris*, *B. lucorum, B. lapidarius*	Δ9	Z9-14:acid, Z9-16:acid, Z9-18:Acid	(Wang et al., 2016)
/	Δ4-Bluc/Bter, Δ4-Blap	*B. terrestris, B. lucorum*, *B. lapidarius*	Δ4	Z4-14:acid	(Wang et al., 2016)
692567	Desat1	*Bombyx mori*	Δ11/Δ10,12 (E,Z)	Z11-16:acid, E,Z-10,12-16:acid	(Riddervold et al., 2002)
23820968	CpaZ9(16>18)	*Choristoneura parallela*	Δ9	Z9-16:acid	(Zhou et al., 2008)
23820970	CpaZ9a(16>18)	*C. parallela*	Δ9	Z9-16:acid	(Zhou et al., 2008)
33335914	CpaZ9(18>16)	*C. parallela*	Δ9	Z9-18:acid	(Zhou et al., 2008)
33335910	CpaZ9(14–26)	*C. parallela*	Δ9	Z9-14:acid, Z9-16:acid, Z9-18:acid, Z9-26:acid	(Zhou et al., 2008)
33335918	CpaE11	*C. parallela*	Δ11	E11-14:acid	(Zhou et al., 2008)
23820962	Cro-Z9(16)	*C. rosacea*	Δ9	Z9-16:acid, Z9-18:acid (16:18≈2:1)	(Wang et al., 2010)
24021793	Cro-Z/E11	*C. rosacea*	Δ11	Z11-14:acid, E11-14:acid (Z:E≈7:1)	(Wang et al., 2010)
562745524	Desat7	*Ctenopseustis herana*	Δ5	Z5-14:acid	(Liu et al., 2002b)
562745522	Desat7	*C. obliquana*	Δ5	Z5-14:acid	(Liu et al., 2002b)
354620210	Desat6	*C. obliquana*	Δ9	Z9-18:acid	(Bucek et al., 2013)
5730151	Tai desat1	*Drosophila melanogaster*	Δ9	Z9-16:acid, Z9-18:acid	(Moto et al., 2004)
1621652	Cs desat1	*D. melanogaster*	Δ9	Z9-16:acid, Z9-18:acid	(Moto et al., 2004)
6735407	Tai desat2	*D. melanogaster*	Δ9	Z9-14:acid	(Moto et al., 2004)
161407181	Dpu-Δ11-APSQ	*Dendrolimus punctatus*	Δ11	Z9,E11-16:acid	(Liu et al., 2004)
161407183	Dpu-Δ11-LPAE	*D. punctatus*	Δ11	Z11-18:acid	(Liu et al., 2004)
161407185	Dpu-Δ9-KPSE	*D. punctatus*	Δ11	Z7,E9-14:acid,	(Liu et al., 2004)
17266299	LBAM-FBZ9	*Epiphyas postvittana*	Δ9	Z9-16:acid, Z9-18:acid (16:18 = 10:7)	(Hao et al., 2002b)
15278212	LBAM-PGZ9	*E. postvittana*	Δ9	Z9-16:acid, Z9-18:acid (16:18 = 1:4)	(Hao et al., 2002b)
16024890	LABM- PGE11	*E. postvittana*	Δ11	E11-14:acid, E11-16:acid	(Hao et al., 2002b)
27151865	HassKPSE	*H. assulta*	Δ9 (C16 > C18)	Z9-16:acid, Z9-18:acid	(Hagstrom et al., 2014)
27151869	HassNPVE	*H. assulta*	Δ9 (C18 > C16)	Z9-16:acid, Z9-18:acid	(Hagstrom et al., 2014)
27151867	HassLPAQ	*H. assulta*	Δ11	Z11-16:acid	(Hagstrom et al., 2014)
8926311	HzPGDs1	*Helicoverpa zea*	Δ11	Z11-16:acid	(Albre et al., 2012)
8926313	HzPGDs2	*H. zea*	Δ9	Z9-16:acid, Z9-18:acid	(Albre et al., 2012)
161376766	Lca-QPAQ	*Lampronia capitella*	Δ9	Z9-14:acid, Z9-16:acid, Z9-18:acid	(Dallerac et al., 2000)
161376772	Lca-KVPQ	*L. capitella*	Δ11	Z11-16:acid, Z,Z-9,11-14:acid	(Dallerac et al., 2000)
/	Lbo_PPTQ	*Lobesia botrana*	Δ11	Z11-14:acid	(Lienard et al., 2010)
162424694	MbraLPAQ	*Mamestra brassicae*	Δ11	Z11-16:acid, Z11-18:acid	(Liu et al., 2002a)
23393788	MdomΔ9	*Musca domestica*	Δ9	Z9-16:acid, Z9-18:acid	(Jeong et al., 2003)
160357984	MsexAPTQ/MsexD2	*Manduca sexta*	Δ11/Δ10,12	Z11-16:acid, E,Z-10,12-16:acid	(Rosenfield et al., 2001; Lienard et al., 2008)
160357986	MsexD3	*M. sexta*	Δ14	Z14-16:acid, E14-16:acid	(Rosenfield et al., 2001)
922667542	MsexD6	*M. sexta*	Δ11	Z11-18:acid	(Rosenfield et al., 2001)
922667544	MsexD5	*M. sexta*	Δ14 (Z/E)/Δ13	Z/E14-16:acid, E13-16:acid	(Rosenfield et al., 2001)
336042963	Obr-LPAQ	*Operopthera brumata*	terminal (ω3)	Z11,Z14,Z17,Z19-20:acid	(Ding et al., 2021)
16797676	Ofu916	*Ostrinia furnacalis*	Δ9 (C16 > C18)	Z9-16:acid, Z9-18:acid	(Park et al., 2008)
16930799	Ofu-Z/E14	*O. furnacalis*	Δ14	Z14-16:acid, E14-16:acid	(Park et al., 2008)
18254516	Ofu-Z/E11	*O. furnacalis*	Δ11	Z11-14:acid, E11-14:acid	(Park et al., 2008)
16797678	Ofu918	*O. furnacalis*	Δ9	Z9-16:acid, Z9-18:acid	(Park et al., 2008)
226423662	LATPG1	*O. latipennis*	Δ11	E11-14:acid	(Eigenheer et al., 2002)
7271960	Onu916	*O. nubilalis*	Δ9	Z9-16:acid, Z9-18:acid	(Park et al., 2008)
20514970	Onu-Z/E14	*O. nubilalis*	Δ14	Z14-16:acid, E14-16:acid	(Park et al., 2008)
16798419	Ofu918	*O. nubilalis*	Δ9	Z9-16:acid, Z9-18:acid	(Park et al., 2008)
20514976	Onu-Z/E11	*O. nubilalis*	Δ11	Z11-14:acid, E11-14:acid (Z:E≈7:3)	(Park et al., 2008)
12025692	Pocto-Z9	*P. octo*	Δ9	Z9-16:acid, Z9-18:acid	(Bucek et al., 2015)
15418735	Pocto-Z10	*P. octo*	Δ10	Z10-16:acid	(Bucek et al., 2015)
KU755471	SexiDes5	*Spodoptera exigua*	Δ11/Δ12	Z11-16:acid, Z9,E12-14:acid	(Matouskova et al., 2007)
AFO38465.1	SexiDes7	*S. exigua*	Δ9	Z9-16:acid, Z9-18:acid	(Matouskova et al., 2007)
AFO38464.1	SexiDes11	*S. exigua*	Δ9	Z9-16:acid, Z9-18:acid	(Matouskova et al., 2007)
111348057	SlitDes11	*S. littoralis*	Δ11	Z11-16:acid	(Ding et al., 2011)
34538646	Sls-FL1(16)	*S. littoralis*	Δ9 (C16 > C18)	Z9-16:acid, Z9-18:acid	(Roelofs et al., 2002)
34538644	Sls-FL2(18)	*S. littoralis*	Δ9 (C18 > C16)	Z9-16:acid, Z9-18:acid	(Roelofs et al., 2002)
1122241107	Sls-FL3	*S. littoralis*	Δ11	Z11-16:acid	(Roelofs et al., 2002)
XP_022827905.1	SlitDes5	*S. litura*	Δ11/Δ12	Z11-16:acid, Z,Z-9,12-18:acid	(Matouskova et al., 2007)
658103	TcasZ9desA	*Tribolium castaneum*	Δ9	Z9-16:acid, Z9-18:acid	(Fujii et al., 2011)
657939	TcasZ9desB	*T. castaneum*	Δ9	Z9-16:acid, Z9-18:acid	(Fujii et al., 2011)
100233160	TcasZ12	*T. castaneum*	Δ12	Z,Z-9,12-16:acid, Z,Z-9,12-18:acid	(Petkevicius et al., 2022)
576636729	TcasD1	*T. castaneum*	Δ9	Z9-16:acid	(Hao et al., 2002a)
576636736	TcasD4	*T. castaneum*	Δ9	Z9-12:acid, Z9-14:acid, Z9-16:acid	(Hao et al., 2002a)
576636738	TcasD5	*T. castaneum*	Δ9	Z9-18:acid	(Hao et al., 2002a)
133926003	Tpi-PGFAD	*Thaumetopoea pityocampa*	Δ11/Δ10,12	Z11-16:acid, E,Z-10,12-16:acid	(Xia et al., 2019)
2708687	T.niΔ9	*Trichoplusia ni*	Δ9 (C18 > C16)	Z9-16:acid, Z9-18:acid (16:18≈1:6)	(Zhang et al., 2019)
4139325	PDesat-TnΔ11Z	*T. ni*	Δ11	Z11-16:acid	(Rodrígueza et al., 2004)
/	Lbo_31670, Lbo_49602	*L. botrana*	Acyl-CoA Oxidase	Z9-12:acid	(Lienard et al., 2010)

Gene IDs were obtained from the NCBI Gene database.

**Table 2:** Functionally characterized fatty acyl reductases involved in insect sex pheromone biosynthesis.

Gene ID/protein accession	Gene name	Species	Functional classification	Product	Reference
520994398	AseFAR	*Agrotis segetum*	fatty-acyl-CoA reductase	Z11-16:OH	(Hagstrom et al., 2013)
299832914	AmFAR1	*Apis mellifera*	fatty-acyl-CoA reductase	C14:0-C22:0 fatty alcohols	(Horne et al., 2010)
444301216	Ban-wFAR	*Bicyclus anynana*	fatty-acyl-CoA reductase	Z9-14:OH	(Haritos et al., 2014)
444301218	Ban-wFAR2	*B. anynana*	fatty-acyl-CoA reductase	16:OH	(Haritos et al., 2014)
33146306	pgFAR	*Bombyx mori*	pheromone gland-specific fatty-acyl reductase	(E,Z)-10,12-16:OH	(Serra et al., 2007)
28572029	Waterproof	*Diptera melanogaster*	fatty-acyl-CoA reductase	C24:-C26:0 fatty alcohol	(Liu et al., 1999)
AGK27745.1	EpFAR	*Ericerus pela Chavannes*	reduce 26-0:(S) CoA	C26:0 fatty alcohols	(Knipple et al., 1998)
379647524	HarFAR	*Helicoverpa armigera*	fatty-acyl-CoA reductase	Z9-14:OH, Z9-16:OH, Z11-16:OH	(Teerawanichpan et al., 2010)
379647522	HasFAR	*H. assulta*	fatty-acyl-CoA reductase	Z9-14:OH, Z9-16:OH, Z11-16:OH	(Teerawanichpan et al., 2010)
121153395	HzeaFAR1	*H. zea*	fatty-acyl-CoA reductase	Z9-14:OH, 16:OH, Z9-16:OH, Z11-16:OH,	(Lienard et al., 2014)
260907981	HvFAR	*Heliothis virescens*	fatty-acyl-CoA reductase	Z9-14:OH, Z9-16:OH, Z11-16:OH	(Teerawanichpan et al., 2010)
379647520	HsFAR	*H. subflexa*	fatty-acyl-CoA reductase	Z9-14:OH, Z9-16:OH, Z11-16:OH	(Teerawanichpan et al., 2010)
262064602	pgFAR-Z	*Ostrinia nubilalis*	fatty-acyl-CoA reductase	Z11-14:OH	(Moto et al., 2003)
262064600	pgFAR-E	*O. nubilalis*	fatty-acyl-CoA reductase	E11-14:OH	(Moto et al., 2003)
453040664	pgFAR	*O. palustralis*	fatty-acyl-CoA reductase	Z9-14:OH, Z11-14:OH	(Moto et al., 2003)
453040662	FAR	*O. latipennis*	fatty-acyl-CoA reductase	Z11-14:OH	(Moto et al., 2003)
453040668	FAR-XIII	*O. scapulalis*	fatty-acyl-CoA reductase	Z11-14:OH	(Jaspers et al., 2014)
453040672	pgFAR	*O. zaguliaevi*	fatty-acyl-CoA reductase	Z11-14:OH	(Moto et al., 2003)
453040660	pgFAR	*O. furnacalis*	fatty-acyl-CoA reductase	Z11-14:OH, Z12-14:OH	(Moto et al., 2003)
453040674	pgFAR	*O. zealis*	fatty-acyl-CoA reductase	Z11-14:OH	(Moto et al., 2003)
695117885	SexpgFAR I	*Spodoptera exigua*	fatty-acyl-CoA reductase	Z11-16:OH	(Jaspers et al., 2014)
939730888	SexpgFAR II	*S. exigua*	fatty-acyl-CoA reductase	Z9-14:OH, Z9E12-14:OH	(Jaspers et al., 2014)
939730865	SlitpgFAR I	*S. littoralis*	fatty-acyl-CoA reductase	Z11-16:OH	(Jaspers et al., 2014)
939730878	SlitpgFAR II	*S. littoralis*	fatty-acyl-CoA reductase	Z9E11-14:OH	(Jaspers et al., 2014)
290782671	pgFAR	*Yponomeuta padellus*	fatty-acyl CoA reductase II	14:OH, Z11-14:OH, E11-14:OH, Z9-14:OH, 16:OH, Z11-16:OH	(Hu et al., 2018)
290782665	pgFAR	*Y. evonymellus*	fatty-acyl CoA reductase II	14:OH, Z11-14:OH, E11-14:OH, Z9-14:OH, 16:OH, Z11-16:OH	(Hu et al., 2018)
296848232	pgFAR	*Y. rorellus*	fatty-acyl CoA reductase II	14:OH, Z11-14:OH, E11-14:OH, Z9-14:OH, 16:OH, Z11-16:OH	(Hu et al., 2018)

Gene IDs were obtained from the NCBI Gene database.

### 2.3 The pathways assembled

**Figure 1:**
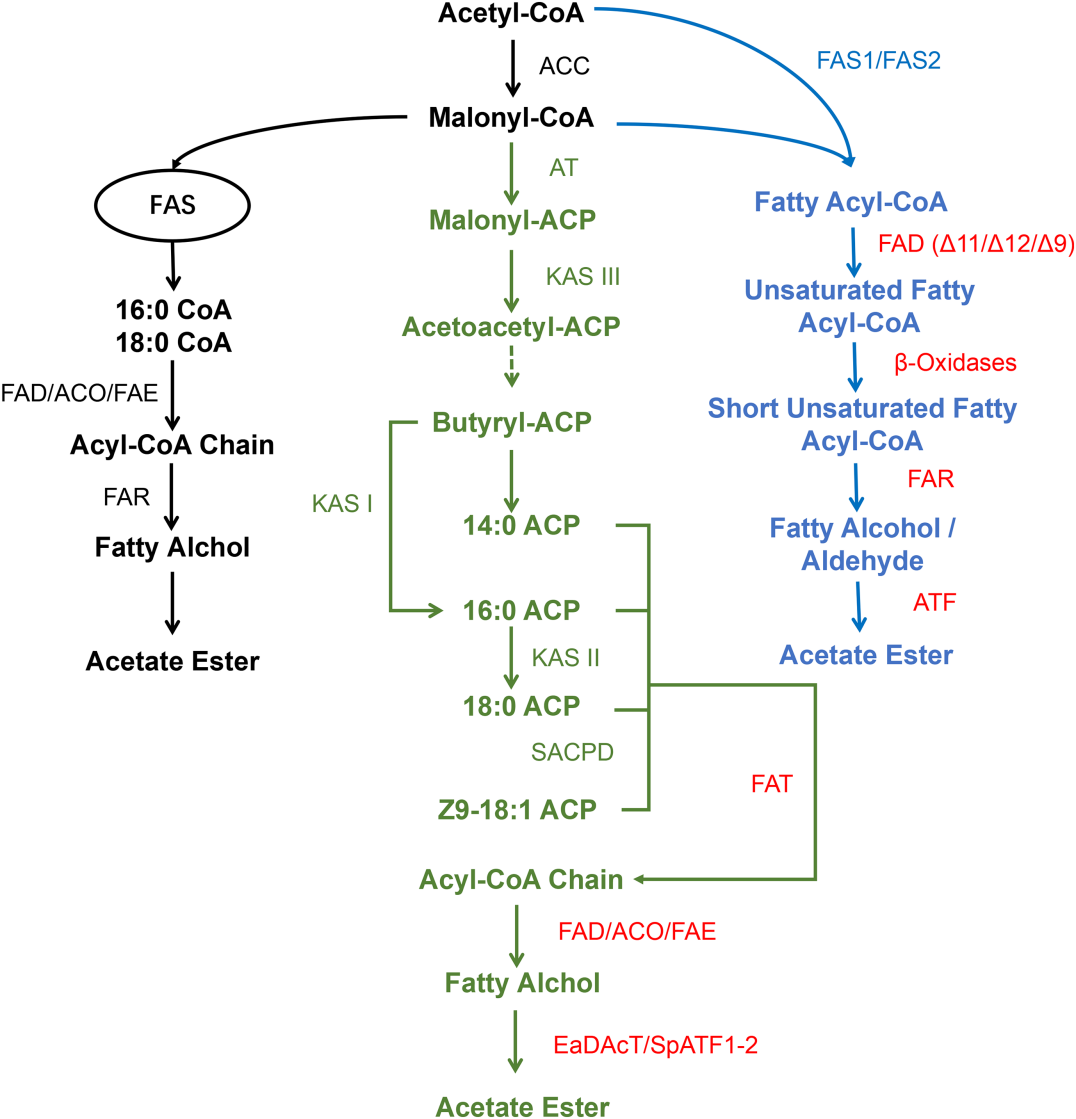
Comparative *de novo* fatty-acid biosynthesis pathways in insects, plants, and yeasts and their metabolic engineering for pheromone precursor production. *De novo* fatty-acid synthesis in insects (black), plants (green), and yeasts (blue) originates from acetyl-CoA and malonyl-CoA via acetyl-CoA carboxylase (ACC) and fatty-acid synthase (FAS), generating intermediates that enter the acyl-CoA pool and downstream lipid metabolism. FAS1/FAS2, fatty-acid synthase subunits 1 and 2; ATF, acetyltransferase; KAS III, β-ketoacyl-ACP synthase III; KASI, β-ketoacyl-ACP synthase I; KAS II, β-ketoacyl-ACP synthase II; SACPD, stearoyl-ACP Δ9-desaturase (a soluble desaturase acting on ACP-linked substrates, primarily in plants); FAT, fatty acyl-ACP thioesterase; FAD, fatty-acyl desaturase (a membrane-bound desaturase acting on CoA-linked substrates, common in insects and animals); FAR, fatty-alcohol reductase; ATF, alcohol acetyltransferase; ACO, acyl-CoA oxidase (catalyzes the first step of β-oxidation); FAE, fatty acid elongase (involved in very-long-chain fatty acid synthesis); EaDAcT, Euonymus alatus diacylglycerol acetyltransferase; SpATF1-2, Sphingomonas sp. alcohol acetyltransferase 1 and 2.ACC initiates fatty acid synthesis; KAS III/I/II extend the carbon chain; FAT hydrolyzes the acyl-ACP thioester bond to release free fatty acids and terminate chain elongation; SACPD (plants) and FAD (insects) introduce double bonds; FAR reduces fatty acids to alcohols; ATF/EaDAcT/SpATF acetylate alcohols to acetate esters (pheromones); ACO and FAE modulate chain length via β-oxidation and elongation, respectively. In plant and yeast systems, engineered or heterologous genes (red) were introduced to reconstruct insect-like pheromone biosynthetic pathways and enhance precursor flux.

Metabolic engineering for pheromone production typically starts from a shared principle: most moth pheromone components derive from fatty-acid metabolism, so improving titers requires reshaping precursor supply (acetyl-CoA/malonyl-CoA and the acyl-CoA pool), minimizing competing sinks and degradation, and then installing the structure-defining enzymes (desaturases, reductases, and, where needed, chain-shortening and acetylation modules). In both yeasts and plants, *de novo* fatty-acid synthesis originates from acetyl-CoA and malonyl-CoA and is initiated by acetyl-CoA carboxylase (ACC) followed by fatty-acid synthase activities; subsequent processing occurs through the acyl-CoA pool and downstream lipid metabolism (**Figure 1**).

#### 2.3.1 Yeast chassis

In yeast, engineering strategies to increase the availability of fatty-acid-derived precursors fall into four recurring categories. First, precursor generation can be enhanced by increasing fatty-acid synthesis and promoting release of free fatty acids, for example by expressing heterologous thioesterases such as a truncated *E. coli* TesA in *Saccharomyces cerevisiae* (Steen et al., 2010). Second, competing activation and utilization routes can be reduced: deletion of FAA1 and FAA4 (major long-chain acyl-CoA synthetases) increases the free fatty-acid pool and can favor downstream conversion to pheromone alcohols (Holkenbrink et al., 2020; Jiang et al., 2022). Third, degradation can be limited by attenuating peroxisomal β-oxidation and related functions (e.g., disruption of acyl-CoA oxidase activity and peroxisome biogenesis factors), while additional deletions of alcohol/aldehyde oxidation routes can reduce loss of alcohol intermediates; combinations of such edits have been reported to substantially improve production of Z11-16:OH in engineered strains (Holkenbrink et al., 2020). Fourth, central carbon supply can be tuned to increase cytosolic acetyl-CoA and NADPH, for example through optimization of citrate/acetyl-CoA generating routes (including ATP:citrate lyase and malic enzyme modules) in oleaginous yeasts (Zhu et al., 2012; Zhou et al., 2016). Finally, for acetate pheromones, acetyltransferase activity can be implemented in yeast using alcohol acetyltransferases (e.g., ATF-family enzymes); interestingly, a yeast-derived acetyltransferase (SpATF1-2) has been reported to provide higher acetylation activity than a previously used plant-derived acetyltransferase in a pheromone context (Ding et al., 2016; Petkevicius et al., 2020).

#### 2.3.2 Plant chassis

In plants, fatty-acid synthesis occurs primarily in plastids, whereas many lipid modifications and acyl-CoA-dependent reactions take place in the endoplasmic reticulum (Lim et al., 2017). Engineering therefore often focuses on redirecting chain-length distribution and expanding the cytosolic/ER acyl-CoA pool that feeds pheromone biosynthetic modules. Introduction of specialized plastidial thioesterases can shift the fatty-acid profile toward shorter-chain products; for example, CpFATB2 from *Cuphea palustris* has been used to increase the availability of C14 substrates in planta, thereby facilitating precursor supply for downstream multienzyme reconstruction of C14-chain pheromone pathways (Ding et al., 2014). With an appropriate precursor landscape in place, pathway reconstruction proceeds by expressing the required desaturases, reductases, and, where applicable, chain-shortening and acetylation modules, while simultaneously reducing competing flux and balancing expression to improve product yield and composition.

### 2.4 The platform alternatives

No single chassis is optimal for all pheromone targets; instead, platform choice is typically guided by (i) the native lipid pool and its capacity for precursor supply, (ii) achievable biomass and productivity, (iii) genetic accessibility and stability of expression, and (iv) how readily the host can support the required functional-group chemistry (e.g., alcohol, aldehyde, acetate).

Yeasts offer rapid design–build–test cycles and well-established tools for pathway integration and flux control. Their endogenous lipid metabolism provides abundant C_16_ and C_18_ acyl substrates, which is advantageous for many moth pheromone backbones. Under nitrogen-limited conditions, oleaginous yeasts accumulate triacylglycerols enriched in C18:1, whereas *S. cerevisiae* typically contains a higher proportion of C16:1 within its TAG fraction, providing a favorable precursor landscape for C16/C18-derived products (Wei et al., 2017). Consistent with this, several yeast systems have been engineered to produce C16- and C18-based pheromone components at measurable titers (Zhang et al., 2021).

Plant-based platforms provide different advantages. High biomass production can enable large-scale, low-cost cultivation, and oilseed crops offer a dedicated storage lipid sink that can stabilize and accumulate hydrophobic products. *Camelina sativa*, for example, is notable for high seed oil content and has been used to accumulate pheromone-relevant fatty-acid intermediates (Bansal and Durrett, 2016; Molina-Hidalgo et al., 2021). However, plant engineering often faces longer iteration cycles, and results obtained in transient expression systems do not always translate directly to stable, field-deployable lines, making the transition from proof-of-concept to production a recurring technical bottleneck (Ding et al., 2014).

Together, these contrasts motivate a modular view of platform selection: yeast systems are well suited to rapid pathway prototyping and optimization, whereas plants—particularly oilseed crops—can offer scalable accumulation of lipid-like intermediates when stable expression and product handling are achieved.

## 3 Technological foundations

**Figure 2:**
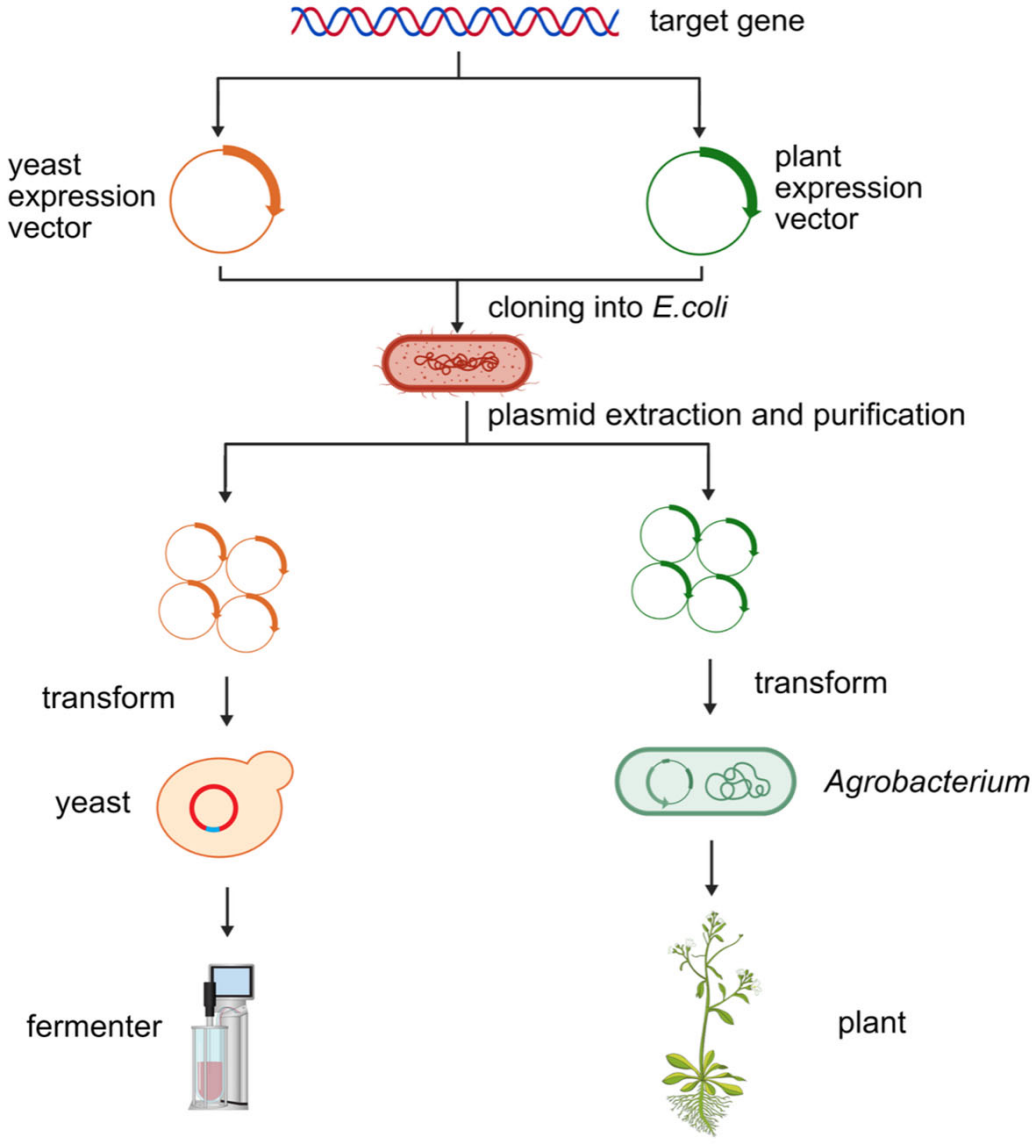
Technical workflow for gene cloning and heterologous expression in multiple hosts. The target gene was cloned into an expression vector and amplified in *Escherichia coli*. In the yeast system (left branch), the plasmid is directly transformed into yeast cells via standard chemical or electroporation methods. In the plant system (right branch), the recombinant plasmid is first introduced into *Agrobacterium tumefaciens*, and the modified *Agrobacterium* is then used to infiltrate plant leaves, enabling transient or stable transformation of plant tissues. Both platforms allow functional characterization of the target gene and production of the corresponding products. The figure is created with BioGDP.com (Jiang et al., 2025).

Biotechnological production of insect pheromones in microbial and plant systems relies on a shared set of synthetic biology capabilities: rapid DNA assembly, efficient genetic delivery, and host engineering to install and tune multi-enzyme pathways. In practice, pathway construction typically follows a common workflow (**Figure 2**): (i) selection and codon-optimization of enzyme modules; (ii) modular cloning and assembly of expression cassettes; (iii) delivery into the chosen chassis and iterative strain/plant line improvement; and (iv) analytical verification of product identity and titers.

### 3.1 Genetic delivery and genome engineering

In plants, *Agrobacterium*-mediated transformation remains a workhorse for both transient expression in leaves and stable transformation in selected species, with particle bombardment providing an alternative where appropriate. An early demonstration of plant-based production introduced an insect Δ11 desaturase via *Agrobacterium* into *Nicotiana* spp., enabling accumulation of (Z)-11-hexadecenoate derivatives that serve as precursors for pheromone components in many moth species. In microbial hosts, CRISPR-based methods are now routinely used to accelerate strain construction, ranging from targeted knockouts to transcriptional regulation of flux-controlling genes. For example, deletion of *FAA1* and *FAA4* in *S. cerevisiae*—genes encoding major long-chain acyl-CoA synthetases—can increase the free fatty acid pool, providing a more favorable precursor landscape for downstream pheromone biosynthesis (Jiang et al., 2022).

### 3.2 DNA assembly standards

Because pheromone pathways often require coordinated expression of multiple genes, vector construction benefits from modular cloning systems. Golden Gate/MoClo and Gateway-based approaches are widely used to assemble multi-gene constructs and to swap promoters, coding sequences, and targeting signals during iterative optimization (Lowder et al., 2015).

### 3.3 Bioprocess development and product analytics

For microbial factories, strain engineering must be paired with fermentation process development, including optimization of medium composition and cultivation parameters (e.g., temperature, pH, aeration), as well as downstream processing to recover and quantify the target product(s). Product identification and quantification are typically performed by GC–MS, with additional techniques such as GC/FT-IR or chiral chromatography used when structural isomers or stereochemistry must be resolved. Collection of plant volatiles can be facilitated by solid-phase microextraction (SPME), with fiber chemistry (e.g., PDMS-based coatings) influencing extraction efficiency; automated formats further improve throughput for screening (Jalili et al., 2020; Xia et al., 2022). Finally, biological relevance is commonly assessed using electrophysiological assays such as GC–EAD and behavioral assays (e.g., wind tunnel tests), which benchmark responses to biologically produced pheromones against synthetic standards (Hagstrom et al., 2013; Petkevicius et al., 2021).

Together, these technological foundations enable rapid build–test–learn cycles for pheromone pathway reconstruction in both microbial and plant chassis. Rather than replacing the need for careful product specification, they provide the experimental infrastructure required to expand enzyme toolkits, improve pathway performance, and systematically compare hosts and designs.

### 3.4 Genetic stability and production durability

For practical implementation, pathway construction must be coupled to genetic stability and long-term production durability. In microbial systems, plasmid-based expression is useful for rapid prototyping but may be compromised during prolonged cultivation by plasmid loss, copy-number variation, and selection against metabolically burdensome pathways (Rouches et al., 2022), whereas genomic integration generally improves stability and reproducibility at the cost of reduced expression flexibility (Xu et al., 2025). In plant systems, stable production is additionally constrained by transgene silencing, positional effects, and fitness penalties associated with constitutive pathway activity (Filipecki and Malepszy, 2006; Singer et al., 2012), particularly when translating transient proof-of-concept results into field-deployable lines (Ding et al., 2014; Xia et al., 2020; Mateos-Fernandez et al., 2021; Kallam et al., 2023). Accordingly, strategies such as genomic integration, copy-number control, dynamic regulation, careful promoter and construct design, and screening for robust elite events are increasingly important for maintaining stable pheromone production over time.

## 4 Case studies of pheromone production in engineered hosts

### 4.1 Microbial systems

A growing number of pheromone biosynthetic enzymes have been functionally characterized, enabling reconstruction of fatty-acid-derived sex pheromone pathways in microbial hosts. Yeasts are particularly attractive for rapid design–build–test cycles and scalable fermentation, and both *S. cerevisiae* and oleaginous yeasts such as *Yarrowia lipolytica* have been developed as production platforms (**Table 3**).

**Table 3:** Metabolic engineering strategies enabling heterologous biosynthesis of insect pheromone precursors in yeast and plant platforms.

Platform	Species	Products	Titers of product	Genetic modification	Highlight strategies	Unintended outcomes	Reference
Yeast	*Saccharomyces cerevisiae*	E10,Z12-16:OH	/	B. mori pgFAR	First cloning of lepidopteran pgFAR; first demonstration of endogenous palmitate utilization; first behavioral bioassay confirming recombinant pheromone activity	Needs exogenous precursor	(Moto et al., 2003)
	*S. cerevisiae*	Z11-16:OH	0.195 mg/L	AseΔ11 (Δ11 FAD) + AseFAR (FAR) from *A. segetum*	Co-expression in single vector; using endogenous yeast palmitate	Low titer; multiple byproducts	(Hagstrom et al., 2013)
	*S. cerevisiae*	Z9,E12-14:OAc	0.32 mg/L	EcaE12 (novel E12 FAD from *E. cautella*) +Dmel_D9(Z9 FAD from *D. melanogaster* + SexipgFARII + yeast ATF1	First doubly unsaturated acetate in yeast; multi-gene assembly; undetectable intermediates; GC-EAD bioactivity confirmed	Behavioral activity and downstream processing not investigated	(Ding et al., 2022)
	*S. cerevisiae*	Z11-16:Ald Z9-16:Ald	22.7 mg/L 45.9 mg/L	Gene knockouts (increase FFA) + Lepidoptera Δ11 FAD + *H. armigera* FAD + OLE1 downregulation + CAR (*M. marinum)*	First CAR (instead of FAR) for aldehyde production; *de novo* synthesis; crude extract bioactive	Titer needs improvement	(Jiang et al., 2022)
	*Yarrowia lipolytica*	Z9-14:OH Z11-16:OH Z9-14:OAc	73.6mg/L 2.57 g/L /	Pathway reconstruction for pheromone precursors; decreased fatty alcohol degradation; downregulated storage lipid accumulation	2.5 g/L titer in bioreactor; field trapping in Greece showed equal efficacy to synthetic pheromone; first demonstration of S. frugiperda acetate production	Requires *in vitro* oxidation for aldehyde production	(Holkenbrink et al., 2020)
	*Y. lipolytica*	Z11-14:OH	188.1mg/L	Fas2p(I1220F) + Lbo_PPTQ + HarFAR + FAS1 OE	FAS engineering; enzyme screening; dosage optimization	Lower bioactivity than synthetic pheromone	(Petkevicius et al., 2021)
	*Y. lipolytica*	Z7-12:OH Z9-12:OH Z7-14:OH	Strain with Dmd9: Z7-12:OH, 0.10 ± 0.02 mg/L Z7-14:OH, 0.48± 0.03mg/L Strain with Lbo_PPTQ: Z9-12:OH, 0.21 ± 0.03 mg/L Z7-14:OH, 0.40 ± 0.07 mg/L	Dmd9 (Δ9 desat from D. melanogaster) or Lbo_PPTQ (Δ11 desat from L. botrana) + HarFAR reductase + Lbo_31670 oxidase + Fas2p(I1220F) mutation	First engineering of β-oxidation in Y. lipolytica for pheromone precursors requiring chain-shortening; peroxidase targeting solved product formation; expanded pheromone portfolio to C12/C14 chain lengths	Low titers (0.10-0.48 mg/L); requires methyl myristate supplementation; Fas2p mutation ineffective; alcohol production only achieved via peroxisomal targeting of reductase	(Petkevicius et al., 2022)
Plant	*Arabidopsis thaliana*	(E)-β-farnesene	483 ng/h	Mentha × piperita Eβf synthase gene	First demonstration of insect pheromone production in transgenic plants; emission of pure Eβf affected both aphids (alarm/repellent) and parasitoids (arrestant) at two trophic levels	Sesquiterpene (not fatty-acid derived); field efficacy later shown to be limited (2015 Rothamsted trial failed to reduce aphid numbers)	(Beale et al., 2006)
	*Camelina sativa*	E9-12:Ac E8, E10-12:Ac	2%-3% in total FA	UcTE (*U. californica* FatB thioesterase) + Cpo_CPRQ (bifunctional Δ9 desaturase from *C. pomonella*) + P19 suppressor; 3 copies of Cpo_CPRQ	First production of moth sex pheromone precursors in engineered oilseed crop; stable in T4; GC-EAD + flight tunnel + orchard trapping confirmed bioactivity	Products are fatty acid precursors	(Xia et al., 2021)
	*C. sativa*	Z11-16:Ac	12.7 ± 1.7% in T6 Seed 19.2 ± 0.4% in T7 Seed	Cuphea FatB (16:0-ACP thioesterase) + AtrΔ11 (*A. transitella)*	First engineering of oilseed crop with FatB + Δ11 desaturase for pheromone precursor; homozygous T4 seeds achieved 22.1% Z11-16:acid; field efficacy equal to synthetic	Precursor requires isolation, purification and conversion to terminal pheromone	(Wang et al., 2022)
	*Nicotiana benthamiana*	Z11-14:Ac E11-14:Ac Z11-16:Ac	54 μg/g 180 μg/g 381 μg/g	14C plant: P19 + CpFATB2 + AveΔ11 + HarFAR + EaDAcT; 16C plant: P19 + AtrΔ11 + HarFAR + EaDAcT	First complete pheromone pathway in plants; first plant acetyltransferase (EaDAcT) for final step; First field validation: Y. padella catches comparable to synthetic; *Y. evonymella* reached 130 males/trap	E/Z ratio not optimal; acetylation requires *in vitro* step; transient expression only; alcohol/acetate ratio control difficult	(Ding et al., 2014)
	*N. benthamiana*	Z11-16:OAc Z11-16:OH	11.8 μg g-1FW 111.4 μg-1 FW	AtrΔ11 desaturase (*A. transitella*) + HarFAR reductase (*H. armigera*) + EaDAcT acetyltransferase (*E. alatus*)	First stable transgenic plants producing volatile moth sex pheromones; volatile release: 8.48 ng/g/day (Z11-16:OH) and 9.44 ng/g/day (Z11-16:OAc); measurable plant-emitted pheromone quantified	Dwarf phenotype correlated with pheromone levels; recurrent EaDAcT truncations in most lines; only 1 line (SxPv1.2) had intact pathway	(Mateos-Fernandez et al., 2021)
	*N. benthamiana*	Z11-16:Alc Z11-16:OH	/	CsupYPAQ (Δ11 desaturase, *C. suppressalis*) + CsupKPSE (Δ9 desaturase, *C. suppressalis*) + CsupFAR2 (*C. suppressalis*) + AtrΔ11 (*A. transitella*) + ATF1 (yeast acetyltransferase)	First demonstration of plant-emitted pheromone aldehydes; first use of yeast ATF1 acetyltransferase in plants; headspace collection of volatiles (SPME) confirmed release of Z11-16:Ald, Z11-16:OH, and Z11-16:OAc	Transient expression only; oxidation mechanism (alcohol to aldehyde) not yet identified	(Xia et al., 2022)
	*N. benthamiana* *N. tabacum;*	Z/E11-14:Ac Z11-16:Ac	/ 335 μg/g	CpuFatB1/CpaFatB2 + AveΔ11/CpaE11/AtrΔ11	First stable vegetative platform for pheromone precursor production; demonstrated *N. benthamiana* as a superior platform for C16 pheromone precursor production	Precursors only; low *N. tabacum* yield; no field validation	(Xia et al., 2020)
	*N. tabacum*	Z11-16:Ac	32 μg/g	TniΔ11 (Δ11 desaturase, *T. ni*)	First GMP for moth pheromone precursors; semi-synthetic conversion to acetate; field-proven attractiveness for M. brassica; green chemistry approach	Precursor only; moderate yield	(Nešněrová et al., 2004)

OE, overexpression. Titers/accumulation values are reported as given in the original studies. Because host systems, cultivation modes, analytical bases, and reporting units differ substantially across studies, these values are intended to provide study-specific performance context rather than strict cross-platform quantitative benchmarking.

Early proof-of-concept studies in *S. cerevisiae* established that introducing insect pheromone enzymes can yield biologically active pheromone alcohols, including E10,Z12-C16:OH following expression of *Bombyx mori* pgFAR (Antony et al., 2009). Subsequent work demonstrated that co-expression of an insect desaturase and FAR could produce common moth pheromone alcohols such as Z11-16:OH at 0.32 mg/L in *S. cerevisiae*, highlighting precursor limitation and competing metabolism as immediate bottlenecks in early pathway engineering efforts (Hagstrom et al., 2013).

Oleaginous yeasts provide a larger native lipid flux and have delivered higher titers after systematic rewiring of precursor supply and degradation. Early heterologous production in *S. cerevisiae* yielded only 0.195 mg/L of Z11-16:OH, which was later improved to 22.7 mg/L through pathway optimization. Subsequent engineering in the oleaginous yeast *Y. lipolytica*, including suppression of fatty alcohol degradation and rewiring of lipid metabolism, further increased production to 2.57 g/L (Holkenbrink et al., 2020). Additional improvements have been achieved by tuning fatty-acid synthesis capacity (e.g., through targeted modifications of FAS and reinforcement of desaturase/reductase modules), reaching improved titers for Z11-14:OH under fed-batch or replenishment cultivation (Petkevicius et al., 2021).

Beyond alcohol products, microbial hosts have also been engineered to access acetate and aldehyde pheromone classes by implementing the required terminal conversions. Introduction of alcohol acetyltransferase activity enables formation of acetate esters from fatty alcohol intermediates, while heterologous carboxylic acid reductases have been used to expand product scope toward aldehydes; in at least one case, crude extracts from engineered yeast exhibited strong attraction to target males in bioassays, underscoring the potential for biologically produced material to retain behavioral activity even prior to extensive purification (Jiang et al., 2022). Collectively, these microbial case studies illustrate a consistent engineering trajectory: initial pathway installation is typically followed by iterative optimization of (i) precursor supply, (ii) suppression of competing sinks and degradation, and (iii) terminal functional-group conversion to reach the desired pheromone class.

### 4.2 Plant-based systems

Plants provide complementary advantages as pheromone production platforms (**Table 4**), including low-cost biomass generation and, in some cases, a dedicated lipid storage sink. Early plant-based demonstrations focused on introducing key insect enzymes into *Nicotiana* to generate pheromone-relevant intermediates, which were subsequently converted to the target acetate by a short downstream processing step and shown to be behaviorally active in field trapping (Nešněrová et al., 2004).

**Table 4:** Comparative features of yeast and plant platforms for insect pheromone biomanufacturing.

Comparative dimension	Yeast platforms	Plant platforms
Carbon source/precursor basis	Defined feedstocks; central carbon metabolism; acyl-CoA pools	Photosynthetic carbon input; fatty acid synthesis; seed oil sinks
Engineering cycle	Rapid DBTL iteration; efficient strain optimization	Fast transient prototyping; slow stable-line development
Expression context	Strong genetic control; membrane-enzyme compatibility constraints	Native eukaryotic context; compartmentation and silencing risks
Product handling	Controlled fermentation; toxicity and recovery constraints	Volatile release in leaves; storage accumulation in seeds
Scale-up logic	Industrial fermentation; predictable process control	Field-scale biomass/seed production; longer deployment timeline
Downstream processing	Extraction and purification often critical	Biomass/seed-based recovery; consistency and stability remain important
Regulatory context	Product-focused regulatory pathway	Additional crop, ecological, and market-acceptance constraints
Current comparative advantage	Rapid optimization; controlled production of defined targets	Scalable precursor accumulation; storage-lipid–based production

Transient expression in *N. benthamiana* enabled rapid testing of multi-enzyme pathways and demonstrated that plants can produce blends of moth pheromone alcohols and acetates that remain attractive and species-specific, even when blend ratios deviate from the natural profile (Ding et al., 2014). This transient leaf-based format is particularly valuable for rapid prototyping, because pathway combinations enzyme variants and subcellular targeting strategies can be tested on short timescales. However, plant pathway performance is strongly influenced by compartmentalization: fatty acid precursors are generated in plastids, whereas many downstream acyl-CoA dependent modifications and terminal conversions occur in the endoplasmic reticulum, creating potential bottlenecks in precursor transfer, enzyme colocalization, and flux matching across compartments. Consistent with this, ER targeting of a fatty acyl reductase increased Z11-16:OH accumulation to approximately 18 μg/g fresh leaf tissue in *N. benthamiana* leaves [18].

Stable transformation has since enabled prolonged production across generations and supports a clearer assessment of how pathway flux and product accumulation affect plant fitness and growth, revealing practical limits when production and/or volatilization becomes excessive (Xia et al., 2020). Compared with transient systems, stable lines are more relevant to field deployment because they allow evaluation of trait stability, inheritance, agronomic performance, and long-term production capacity. However, they also introduce longer development cycles, stronger selection for expression instability or silencing, and greater sensitivity to growth penalties associated with constitutive or high-level pathway activity [34].

Oilseed crops offer an alternative strategy in which pheromone-relevant intermediates are accumulated in seed oil. *C. sativa* has been engineered to produce codling moth pheromone precursors such as E9-12:Ac and E8,E10-12:Ac at levels of approximately 2–3% of total fatty acids, demonstrating the feasibility of stable, storage-lipid-based accumulation (Xia et al., 2021). Importantly, Camelina-produced precursors for pheromone components have been evaluated in field applications and reported to perform comparably to synthetic standards in pest control, indicating that biological production can deliver functional material for deployment under realistic conditions (Wang et al., 2022). More recent efforts have explored co-expression of desaturases, reductases, and heterologous acetyltransferase modules (e.g., yeast ATF1) to enhance acetate formation and/or volatilization *in planta*, highlighting the value of importing terminal conversion activities when endogenous activities are insufficient (Xia et al., 2022).

Together, plant-based case studies highlight two recurring themes: (i) transient leaf expression is well suited to rapid pathway prototyping and product profiling, whereas (ii) stable production, especially in oilseed platforms, better supports accumulation, storage, and scale-up, but introduces longer development cycles and fitness-related constraints that must be managed. More broadly, engineering predictability is currently lower in plants than in microbial systems, because pathway output is more strongly shaped by tissue context, compartmentalization, developmental regulation, and transgene stability. These features make plant systems highly attractive for scalable production and deployment, but often less straightforward to optimize in a fully designable manner than microbial chassis.

To place these complementary features into a common framework, the major comparative characteristics of yeast and plant platforms are summarized in **Table 4**.

## 5 Economic and environmental impact

### 5.1 Cost-effectiveness

Industrial pheromone supply is still dominated by chemical synthesis, which often requires multi-step routes and careful control of double-bond position/geometry and stereochemistry. These requirements can increase process complexity and influence cost, particularly for blends that demand high isomeric purity (Zhang et al., 2013; Turczel et al., 2018).

Bio-based production in engineered microbes or plants offers an alternative manufacturing logic. Rather than constructing the carbon backbone through stepwise chemical transformations, biological platforms generate pheromone precursors from central carbon metabolism and lipid pathways. In principle, this may reduce dependence on some specialized synthetic steps and simplify access to certain intermediates, but the extent of any practical advantage remains product- and process-dependent. As illustrated in the previous sections, engineered microbial systems have enabled production of moth pheromone precursors and alcohols directly from central carbon and lipid metabolism, with reported titers ranging from sub-mg/L in early proof-of-concept systems to gram-per-liter scale in optimized yeast strains (Hagstrom et al., 2013), highlighting their potential for scalable fermentation-based production. In this context, microbial systems may be most competitive for products that benefit from tightly controlled fermentation, rapid strain optimization, and relatively straightforward downstream purification (Holkenbrink et al., 2020; Petkevicius et al., 2020). By contrast, plant systems may be advantageous when pheromone intermediates can be accumulated at scale in biomass or seed oil, particularly when precursor-based production is sufficient and low-cost field-scale production can offset longer development cycles (De Martinis et al., 2022).

However, current evidence remains insufficient to support broad quantitative claims about cost advantage across platforms or products. Published studies differ substantially in target molecules, host systems, production scales, and analytical boundaries, and many do not report comparable techno-economic assumptions (Yang et al., 2022). As a result, competitiveness must still be evaluated on a case-by-case basis. In practice, economic performance is likely determined most strongly by three factors: product titer, downstream purification burden, and blend complexity. High titers are important, but their benefit may be offset if purification must resolve closely related isomers or remove substantial by-product backgrounds. Similarly, multi-component pheromone blends may remain costly even when individual components can be produced efficiently, because achieving the correct blend ratio and stereochemical purity can dominate overall process economics. Thus, rigorous techno-economic analyses (TEA) across representative products and production formats are still needed before broad quantitative generalizations can be made.

### 5.2 Environmental benefits

Pheromone-based strategies can reduce dependence on broad-spectrum insecticides by shifting control toward behavior-based interventions, including mating disruption and attract-and-kill approaches. In orchard systems, field studies have reported that mating disruption can reduce the number of insecticide applications substantially while maintaining or improving control outcomes; for example, one two-year commercial-conditions study on codling moth in apple orchards reported a ~44-45% reduction in codling moth–targeted sprays in mating-disruption orchards compared with conventional orchards (Kutalmis and Ogur, 2025).

Because pheromones are species-specific and used at very low per-hectare application rates, they are generally considered compatible with conservation of beneficial arthropods and pollinators, although actual ecosystem outcomes depend on the broader IPM program and local management context. More broadly, concerns about non-target impacts of some insecticide classes, including evidence of negative effects of neonicotinoid exposure on wild bees in agricultural landscapes, have strengthened interest in complementary, lower-impact control tools (Woodcock et al., 2016).

### 5.3 Sustainability

From a sustainability perspective, bio-based pheromone manufacture offers two potential advantages: renewable carbon inputs and the possibility of integrating production into existing bioprocess or crop systems. Microbial fermentation can in principle use a broader range of feedstocks than purified sugars alone. For example, several studies reported glycerol-fed *de novo* production of several Type I pheromone alcohols in *Y. lipolytica*, including Z11-16:OH, Z11-14:OH and shorter-chain products (Hu et al., 2020; Petkevicius et al., 2022), and also present pheromone precursor production from pure glycerol in *E. coli* (Wu et al., 2014) and from corn stover hydrolysate in engineered *S. cerevisia*e (Li et al., 2023); *R. toruloides* has likewise been reported to produce PUFA precursors from galactose/raffinose mixtures (Wang et al., 2016). These examples support the feasibility of carbon-source flexibility in microbial systems, although their environmental and economic significance remains case specific. In contrast, plant-based systems can directly exploit photosynthetically fixed carbon and, in oilseed platforms, a storage-lipid sink that is well matched to hydrophobic pheromone intermediates. For example, engineered *C. sativa* seeds have been used to accumulate the pheromone precursor Z11-16:acid for downstream conversion and field validation (Wang et al., 2022).

Nevertheless, sustainability claims must be interpreted cautiously and evaluated on a product- and process-specific basis. Key determinants include land use and agronomic inputs for plant platforms, and energy demand and solvent use, and downstream separation requirements for microbial platforms. A clear priority for the field is therefore to develop comparative life-cycle assessments (LCA) and TEA using harmonized assumptions, enabling transparent benchmarking against conventional chemical synthesis routes and identifying the main environmental “hot spots” for improvement.

Finally, adoption at scale also depends on regulatory and social acceptance. For example, the use of transgenic crops as production platforms may face policy and market constraints in some regions, whereas microbial production must reach sufficiently high titers and robust downstream processing to meet industrial specifications. These considerations reinforce the need to align platform choice and product design with both technical feasibility and deployment realities.

## 6 Future directions and innovations

### 6.1 Research gaps

Across both microbial and plant platforms, successful case studies converge on a common design logic: pathway installation is rarely sufficient on its own. Useful titers and acceptable product spectra typically require coordinated optimization of precursor supply (acetyl-CoA, malonyl-CoA and acyl-CoA pools), suppression of competing sinks and degradation, and careful selection of enzyme variants that deliver the required regio-/stereoselectivity. Platform-specific constraints then shape optimization priorities: in microbes, these often center on bioprocess conditions, heterologous expression of membrane-bound enzymes, cofactor and electron-transfer compatibility, and toxicity of pathway intermediates or products; in plants, key limitations more often include compartmentalization, transgene silencing, expression instability across generations, and trade-offs between volatile production, growth, and fitness.

These shared patterns also clarify why predictable, scalable pheromone biomanufacturing remains challenging. Three gaps are particularly limiting: (i) toolkit coverage and selectivity, as available desaturases/reductases do not yet provide sufficient regio-/stereo control for many targets and terminal conversions to aldehydes/acetates remain uneven across hosts; (ii) host context dependence, because enzyme performance and product spectra vary with acyl-CoA pools, redox supply and competing lipid pathways, so “parts lists” rarely transfer across chassis without re-optimization; and (iii) benchmarking, since the lack of standardized build–test conditions and harmonized analytics hampers cross-study comparisons of titer, selectivity and by-product profiles. Together, these insights provide a practical framework for host selection and for prioritizing toolkit and workflow improvements for new pheromone targets.

### 6.2 Emerging technologies

CRISPR-based genome engineering enables multiplex rewiring of lipid metabolism (precursor supply, competing sinks, β-oxidation, and dynamic control), accelerating iteration in both microbes and plants. Data-driven approaches may further improve design by prioritizing enzyme variants and guiding pathway balancing, but their impact will depend on curated, comparable datasets generated under standardized workflows.

### 6.3 Commercial bioproduction of pheromones

Industrial pheromone production is currently supported by several distinct manufacturing routes. Traditional chemical synthesis remains the dominant platform and is still used by major suppliers such as Shin-Etsu, BASF, Suterra, Bedoukian Research, and ISCA. In parallel, biologically enabled production routes are beginning to reshape the field. Microbial fermentation, pioneered by BioPhero and further advanced after its acquisition by FMC in 2022, uses engineered yeast to manufacture pheromones at lower cost and is explicitly aimed at expanding deployment into row crops such as corn and soy. Provivi represents another important commercialization route, combining proprietary low-cost manufacturing with greener synthetic processes and bio-based raw-material integration to make pheromone products more accessible for broad-acre crops. In China, Pherobio Technology (Zhongjiesifang) has developed a broad pheromone and semiochemical portfolio and reports sales in more than 50 countries, reflecting the growing internationalization of pheromone-based crop protection.

### 6.4 Policy and regulation

Regulatory requirements for pheromone products and for engineered production platforms vary across jurisdictions. Clear standards for composition and quality control, together with transparent risk assessment and pilot-scale validation pathways, will be important for wider adoption of biologically produced pheromones.

In summary, progress will likely be driven by expanding enzyme toolkits, improving cross-platform predictability through standardized benchmarking, and deploying modern genome engineering and data-driven methods to address recurring metabolic bottlenecks.

## 7 Conclusion

Fatty-acid-derived moth sex pheromones are defined by a small set of biochemical decision points—chain length, double-bond position/geometry, and terminal functional groups—yet practical production requires enzyme modules that deliver these features with high selectivity and predictable performance. In this review, we organized the field around these constraints and summarized the current gene pool and synthetic biology toolkit enabling pathway reconstruction in yeast and plant platforms.

Case studies across microbial and plant systems show that biologically produced pheromone intermediates and blends can be achieved through iterative, modular engineering that couple’s pathway installation with systematic rewiring of host lipid metabolism. However, broader translation is still limited by incomplete enzyme coverage and regio-/stereoselectivity, host context dependence, and a lack of standardized benchmarking across studies and chassis.

Future progress will therefore rely on expanding and engineering enzyme toolkits, improving cross-platform predictability through harmonized build–test–measure workflows, and applying modern genome engineering and data-driven design to address recurring precursor, flux, and product-toxicity constraints. Taken together, these developments suggest that microbial and plant “biological factories” to become increasingly useful complements to conventional synthesis for selected pheromone targets, although their large-scale applicability will depend on further technological and process optimization.
